# A Study on the Failure Behavior of Sand Grain Contacts with Hertz Modeling, Image Processing, and Statistical Analysis

**DOI:** 10.3390/s21134611

**Published:** 2021-07-05

**Authors:** Siyue Li, Sathwik S. Kasyap, Kostas Senetakis

**Affiliations:** Department of Architecture and Civil Engineering, City University of Hong Kong, Hong Kong, China; siyueli6-c@my.cityu.edu.hk (S.L.); ssarvadev2-c@my.cityu.edu.hk (S.S.K.)

**Keywords:** proppant, particle crushing, failure mode, Hertz modeling, local radius, image processing, clustering analysis

## Abstract

The crushing behavior of particles is encountered in a large number of natural and engineering systems, and it is important for it to be examined in problems related to hydraulic fracturing, where proppant–proppant and proppant–rock interactions are essential to be modeled as well as geotechnical engineering problems, where grains may crush because the transmitted stresses at their contacts exceed their tensile strength. Despite the interest in the study of the crushing behavior of natural particles, most previous experimental works have examined the single-grain or multiple-grain crushing configurations, and less attention has been given in the laboratory investigation of the interactions of two grains in contact up to their failure as well as on the assessment of the methodology adopted to analyze the data. In the present study, a quartz sand of 1.18–2.36 mm in size was examined, performing a total of 244 grain-to-grain crushing tests at two different speeds, 0.01 and 1 mm/min. In order to calculate stresses from the measured forces, Hertz modeling was implemented to calculate an approximate contact area between the particles based on their local radii (i.e., the radius of the grains in the vicinity of their contact). Based on the results, three different modes of failure were distinguished as conservative, fragmentary, and destructive, corresponding to micro-scale, meso-scale, and macro-scale breakage, respectively. From the data, four different classes of curves could be identified. Class-A and class-B corresponded to an initially Hertzian behavior followed by a brittle failure with a distinctive (single) peak point. The occurrence of hardening prior to the failure point distinguished class-B from class-A. Two additional classes (termed as class-C and class-D) were observed having two or multiple peaks, and much larger displacements were necessary to mobilize the failure point. Hertz fitting, Weibull statistics, and clustering were further implemented to estimate the influence of local radius and elastic modulus values. One of the important observations was that the method of analysis adopted to estimate the local radius of the grains, based on manual assessment (i.e., eyeball fitting) or robust Matlab-based image processing, was a key factor influencing the resultant strength distribution and *m*-modulus, which are grain crushing strength characteristics. The results from the study were further compared with previously reported data on single- and multiple-grain crushing tests.

## 1. Introduction 

Granular materials are encountered in numerous applications such as sedimentology and geophysics, geotechnical and mining engineering, and energy and industrial applications. Understanding the interactions between grains has been a topic of major interest in the scientific community as the load transfer mechanisms, flow behavior, breakage of particles, and changes in the fabrics of granular assemblies [1,2,3,4,5], as well as their frictional response [6,7], control the bulk strength and deformation characteristics as well as the energy dissipation mechanisms of granular systems.

In petroleum engineering, developments in unconventional reservoir technologies and hydraulic fracturing have led to an increased interest in the study of the interactions of proppants, including their damage behavior [8,9] and the frictional response between proppants and rocks [10,11,12]. These problems involve, among other aspects, grain global crushing and embedment of the proppants, which influence fluid flow and the quality of the production process. In many of the above-mentioned studies, the examination of the interaction of interfaces (at pre-failure or failure regimes) necessitates the implementation of advanced sensing and measurement techniques, for example, high-precision sensors or image process techniques may be important for an accurate investigation of these problems that involve minute displacements and the influence of surface morphology. The interactions of particles and their frictional and breakage behavior have also been topics of major interest in geomechanics research as, apart from the fundamental study of the behavior of soils and rocks, for example, the development of strong force chains/load transfer mechanisms and fabric evolution [13,14,15], particle breakage is a key aspect to understand creep-relaxation mechanisms in granular systems [16], their compression and shearing behavior [5,16,17,18], and also soil–structure interaction problems [19]. 

In their recent study, Sandeep et al. [20] quantified the influence of size (or scale) and morphology effects on the contact behavior of granular materials using benchmark glass beads and a particle-to-particle configuration that allowed the examination of the behavior of grains at their contacts. It was shown in that study, based on analysis using high-precision sensors and nanometer-level measurements of the topological characteristics of the grains, that surface roughness is a dominant factor in controlling both the normal contact behavior and the frictional response of granular materials, and they attempted to link the microscopic observations from their work with previously published data that reported on the bulk behavior of granular materials. That study focused on the behavior of granular materials at relatively lower confining pressures (or contact forces), well below the level that causes global grain breakage. The influence of grain type and meso-scale morphological features (e.g., the shape of the grains in the vicinity of their contacts) was examined in recent studies by Wang and Coop [21] and Todisco et al. [22], attempting to investigate single- and multiple-contact configurations of particles. For example, based on single-particle crushing tests on quartz-type grains and the use of a high-speed camera, Wang and Coop [21] compared the breakage strength of particles that had “splitting” and “explosive” modes of failure (as two major types of observed crushing modes); the former mode was ascribed to particles that developed a crack at failure and perhaps the grain was split into two parts, while the latter mode was ascribed to particles that were fragmented in many pieces with an explosive failure. Based on their study, the mean strength of the grains (fraction 1.18–2.36 mm) with splitting and explosive modes were approximately equal to 38 and 46 MPa, while a decrease of the mean strength of the order of (approximately) 25% and 17% was observed for splitting and explosive modes of LBS grains, fraction 2.36–5.00 mm.

Earlier works provided important insights on the behavior of grains with respect to the onset of breakage, however, most works attempted to examine single-grain configurations, which involve the compression of a particle from top and bottom stiff blocks (or platens). This configuration, even though it provides a basic characterization of individual grains, is far from the way grains interact in real assemblies, which develop contacts among various particles and form strong and weak force chains. The study by Wang and Yan [4] using discrete-based numerical simulations attempted to examine the energy dissipation mechanisms in crushable materials. Based on their results, it was shown that grain crushing itself was not a major contributing mechanism in energy dissipation but led to the increase of the contacts between particles, which in turn mobilized an important energy dissipation mechanism associated with inter-particle friction. 

Despite the significant developments in the study of granular materials, both experimentally and numerically, the problem of the interaction of two grains at their contact with an emphasis on the onset of grain breakage has been heavily overlooked, and most researchers have either worked on the problem of grain contact response generally at much lower confining loads [23,24,25], or the breakage behavior of particles has been studied, predominantly, by means of single-grain crushing tests [26,27,28] as well as through observations of the behavior of grain assemblies, for example, element-sized samples [29,30,31], or through discrete-based computational analysis [4,5,32,33]. Particularly in the discrete-based simulation of the grain crushing and its evolution in granular assemblies, researchers need accurate experimental data to use as input; thus, the precise analysis of the grain crushing behavior and the critical review of the methods of analysis are important to be assessed experimentally.

Insights into the crushing behavior of microparticles, in which microparticles formed debris in the vicinity of sand grain contacts, were obtained in the recent study by Kasyap et al. [34]. They reported that the crushing of microparticles in the vicinity of the sand grain contact heavily controlled the frictional response of the granular system and led to different prevalent mechanisms depending on the type of microparticles (which in turn influenced their crushing behavior). An important mechanism contributing to the frictional behavior of grain contacts was also observed by Sandeep and Senetakis [35] during a study in which meso-scale crushing of asperities and formation of debris during the compression and shearing processes of the grains led to a major increase in the tangential stiffness as observed in subsequent loading cycles. Despite these insights, the above-mentioned studies also focused on relatively small confining forces (or normal forces) that are well below the magnitude that would cause global crushing of the sand grain system.

Based on the literature gaps in the examination of the interactions of grains at their contacts, particularly in grain-to-grain configurations, as well as the critical assessment of the methods of analysis and how they influence the interpretations from grain-scale tests, it was attempted in the present study to investigate the contact behavior of sand grains in the form of grain-to-grain systems applying compression loads up to the failure of the specimens. As these experiments involve grain contacts, the Hertz model [36] was applied to estimate the contact area, which is necessary to be obtained to derive stresses from the measured forces. The application of Hertz modeling has been implemented in many previous studies examining the contact behavior of particles (e.g., [25,37,38]), however, this has been majorly directed in the analysis of the pre-failure behavior of granular systems, which was one of the major novel concepts in the present study, i.e., implementation of Hertz analysis in grain contact crushing experiments. An important new contribution from the present work was also the rigorous examination of the influence of the method of analysis of the data, particularly the way local radius (i.e., the radius of the grains in the vicinity of their contact) is estimated to apply Hertz modeling. For this purpose, image processing was implemented based on a custom-written Matlab code in order to assess the local shape of the grains in the vicinity of their contacts, and the results were critically compared with other available methods. Note that the local morphology of the grains was assessed prior to setting in contact of the two particles, which is the common method in assessing the influence of particle characteristics on the behavior of granular materials (both in terms of particle-scale and macroscopic behavior). However, advanced techniques such as X-ray micro-CT scanning can be used as an alternative method to tackle the changes in particle local morphology during the increase of the confinement, which were not available in the authors’ laboratory. 

## 2. Methodology

### 2.1. Materials, Experimental Setup, and Testing Program

The crushing tests were performed in a modified CBR apparatus that is capable of testing the strength of grains in different configurations, such as single-grain breakage tests or multiple-contact breakage tests (after [21,22,27]). In the present study, the strength of grain-to-grain systems was examined using Leighton Buzzard sand (LBS) particles of approximately 1.18 to 2.36 mm in size. LBS consists of quartz-type sand with particles that have a golden yellow to brown color with a fairly spherical and subrounded to rounded shape, and it has origins from the UK. This material has been used in previous studies in both crushing tests [21,22,27] as well as frictional tests by means of grain-to-grain tribological experiments [25,39] and it is considered as a proppant simulant (despite its slightly larger size compared with typical fractions used in hydraulic fracturing). Sandeep and Senetakis [24,40] reported values of surface roughness of 223 ± 61 nm based on measurements of the mean square root of peaks from the average value (RMS) roughness using an optical surface profiler, while micro-indentation tests have indicated an average hardness value of 4.9 GPa for LBS grains.

**Figure 1:**
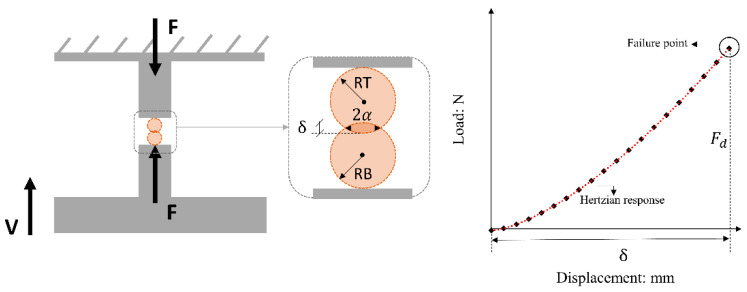
Schematic representation of crushing test setup and representative force—displacement curve.

A schematic configuration of the experimental setup is given in Figure 1. The grains were glued on top and bottom mounts fixed on the vertical loading system and base of the apparatus, respectively. Sufficient time, typically between 24 and 48 h, was allowed so that the glue would harden enough without influencing the grain contact response (after [41,42]). A vertically positioned load cell with a capacity of 1 kN and a precision of ±0.4 N and a displacement sensor with a precision of ±0.2 μm were used to record the vertical (or normal) force and displacement, respectively, and the data were transferred to a data logger for further processing. A total of 244 grain-contact crushing tests were performed in a displacement-controlled mode till the occurrence of failure; 122 tests were carried out at a loading rate (or speed) of 0.01 mm/min and 122 tests were carried out at a speed of 1 mm/min. For all these experiments, the local radius of both upper and bottom grains was assessed using two orthogonally placed digital microscope cameras. The estimation of the local radius in the vicinity of the contacts of the grains was important to be obtained so as to apply Hertz modeling to the experimental vertical load–displacement data and also to calculate the contact area (thus deriving stresses from the measured forces).

A summary of the tests and respective data analyses is given in Table 1. The analysis of contact radius was performed in two different ways; in the first method, the local radius was assessed from visual observations and approximate drawing of “circles” in the obtained images from the microscope cameras (termed as “manual method”), and in the second method the local radius was assessed based on image processing techniques using a custom-written Matlab code (termed as “Matlab method”). Both methods led to the estimation, through the application of Hertz modeling, of the contact radius, which is a key parameter to be obtained for further processing of the data to derive stresses. The analytical expressions used in the data analysis are summarized in Section 2.2. A detailed discussion on the procedures adopted for the “manual method” and ‘Matlab method” for estimating the local radius is presented in Section 3.1 using a representative sample.

### 2.2. Analytical Method

**Figure 2:**
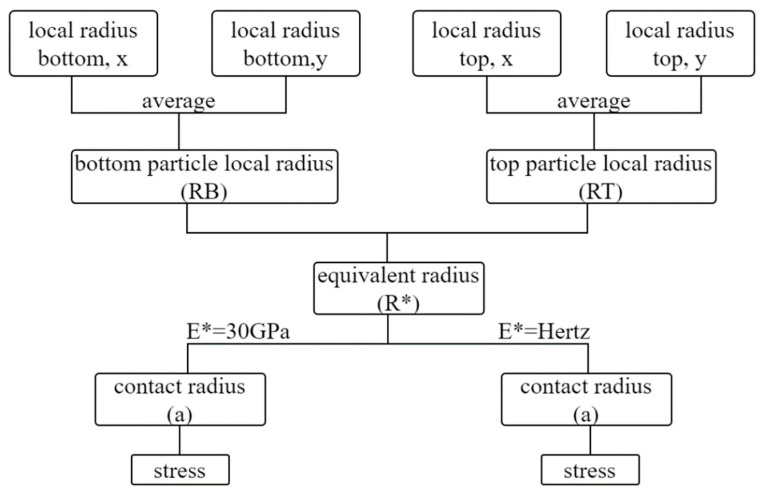
Flowchart of methodology in assessing the contact radius and stress at failure.

A flowchart of the methodology used to compute stresses from the measured forces is given in Figure 2.

Two orthogonally placed digital microscopic cameras were used to capture images of the top and bottom grains from two different directions (termed as “x” and “y” directions corresponding to the “yz” and “xz” planes, respectively, where “z” is the vertical axis). For each grain of the system (i.e., for the top and bottom grains), the arithmetic mean of the local radius as obtained from the two digital cameras was computed, termed as *RB* for the bottom grain and *RT* for the top grain, based on Equation (1a) and (1b), respectively. Subsequently, applying the Hertz model, the equivalent radius (R*) was estimated based on Equation (2), and the contact radius (*α*) was calculated from Equation (3). Based on the estimated contact radius, the stress at failure was calculated based on Equation (4), where *F_d_* is the vertical force at failure (note that a discussion on the failure force through representative force–displacement curves from the experiments is presented in Section 3.5).

(1a) $$RB=\frac{\left(RB_{\mathrm{yz}}+RB_{\mathrm{xz}}\right)}{2}$$

(1b) $$RT=\frac{\left(RT_{\mathrm{yz}}+RT_{\mathrm{xz}}\right)}{2}$$

where *RB* and *RT* are the local radii of the bottom and top particles, respectively, while *RB*_yz_, *RB*_xz_, *RT*_yz_, and *RT*_xz_ were obtained from the images of bottom and top grains from two different directions.

(2) $$\frac{1}{R^{*}}=\frac{1}{RB}+\frac{1}{RT}$$

(3) $$\alpha ={\left(\frac{3F_{d}R^{*}}{4E^{*}}\right)}^{\frac{1}{3}}$$

(4) $$\sigma =\frac{F_{d}}{\pi \alpha^{2}}$$

In Equations (2)–(4), R* is the equivalent radius of the grains, (*α*) is the radius of the circular contact area (computed based on the assumption of Hertzian contact response), *F_d_* is the peak load or failure load during the crushing test, and *σ* is the maximum tensile stress.

E* in Equation (3) is the equivalent (or contact) Young’s modulus, which is obtained from Equation (5).

(5) $$\frac{1}{E^{*}}=\frac{1-\nu_{B}^{2}}{E_{B}}+\frac{1-\nu_{T}^{2}}{E_{T}}$$

where *E_B_*, *E_T_* and *ν_B_*, *ν_T_* are the Young’s moduli and Poisson’s ratio values of the bottom and top particles.

Note that LBS, as natural grains, display some variabilities in their morphological and elastic characteristics and the resultant contact modulus depends on the specific pair of grains (i.e., there is some influence of the surface roughness and meso-scale morphology as well as the specific composition of each grain). Based on the studies by Sandeep and Senetakis [24,25], an E* value of 30 GPa could be considered as “representative” for LBS grain contacts. Thus, after the estimation of the equivalent radius, the derivation of the stress at failure from Equations (3) and (4) was performed in two different ways: (i) all the pairs of grains (referred to as specimens) were assumed to have E* = 30 GPa and this analysis was adopted for both speeds used in the study, thus for the total set of 244 tests, and (ii) E* values were estimated for each individual specimen based on the initial part of the force–displacement curves, thus each given pair of grains displayed its own contact Young’s modulus. The latter analysis was carried out only for the 122 tests performed at 0.01 mm/min so that a sufficient number of experimental data could be used for a reliable Hertz-fitting implementation (Table 1). 

## 3. Results and Discussion

### 3.1. Analysis of Local Radius and Contact Radius

**Figure 3:**
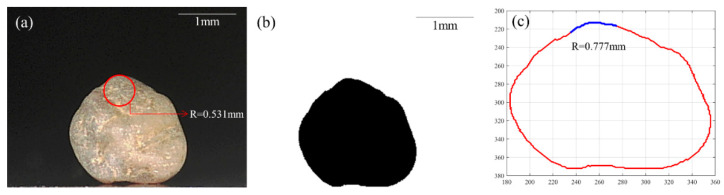
Example of the application of two different methods (Matlab and manual) to obtain the local radius. (**a**) Manual method from image of a grain with a circle fitted to grain boundary, (**b**) Binary image of the grain, (**c**) Pixelated boundary of the grain and the range of boundary used for local radius calculation in Matlab method.

As discussed in Section 2.1, two methods (manual- and Matlab-based) were used to estimate the local radius of the contacting LBS grains. The manual method used in this study is a straightforward technique of eyeball fitting the grain shape with a circle in the vicinity of the contact. The radius of the best-fit circle indicates the local radius of the grain. A similar approach was also adopted in previous micromechanical works (e.g., [25,42]) to estimate the local radius of sand grains, which is different from the global radius (neither circumscribing nor inscribing circle radii). On the other hand, the Matlab method uses the pixelated boundary of the grains to find the statistically best-fit polynomial (fourth-order) for the data points in the required range of the grain boundary. The pixelated boundary of a grain is obtained from the binary image of the original RGB image of the grain, thresholded using a commercial software called ImageJ. The summation of the radius of curvature at each data point on the boundary within the specified pixel range results in the local radius of curvature of a grain. A similar approach was also adopted by Kasyap and Senetakis [37] and Wang and Coop [21] in their micromechanical studies. The procedures of the manual and Matlab methods are further explained using a representative picture of a grain in Figure 3. Figure 3a shows the original image of a grain (RGB color) positioned on the apparatus prior to the crushing test. According to the aforementioned manual method, a circle (shown in red: Figure 3a) is fit to the grain peak where the other grain of the specimen is in contact during the test, and the radius of the circle was measured to be 0.531 mm for the given example. Figure 3b shows the binary image (8-bit after thresholding gray values) of Figure 3a. In the Matlab method, this binary image is processed with image processing tools to obtain the boundary of the grain in terms of their pixel positions (Figure 3c), thereby calculating the local radius of curvature for the selected zone (blue part in Figure 3c). For the example shown in Figure 3, the Matlab method resulted in a local radius of 0.777 mm, 1.5 times higher than the manual method. Similar analyses were conducted on the image of a grain captured from an orthogonal direction in the horizontal plane and the arithmetic mean of the local radii obtained from each direction was considered as the local radius of the grain.

**Figure 4:**
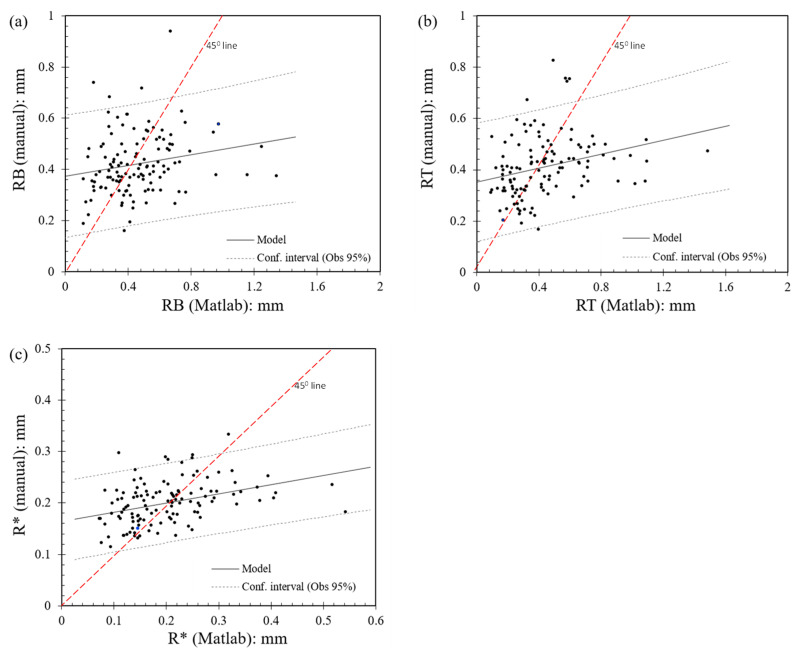
Comparison of (**a**,**b**) local radii of bottom and top grains and (**c**) equivalent radii based on Matlab and manual analysis of local morphology of the grains (experiments at 0.01 mm/min speed).

In Figure 4, a comparison of the estimated values of the local radii for bottom grains (Figure 4a) and top grains (Figure 4b) based on Matlab and manual analyses, as well as the estimated values of equivalent radii from Matlab and manual analyses (Figure 4c) are presented. A correlation coefficient of the data in each of the plots in Figure 4 was estimated. The Pearson correlation coefficient was used to estimate the closeness of the values obtained from Matlab and manual methods with a linear function. At 0.01 mm/h loading rate, *RB*, *RT*, and R* values showed a weak correlation coefficient of 0.20, 0.28, and 0.38, respectively. The correlation coefficients, however, indicate that the manual and Matlab methods are positively correlated. Moreover, another comparison between the *RB*, *RT*, and R* values from the manual and Matlab methods was made by estimating the scatter of data points around the 45° line in Figure 4. Out of 121 data points in each plot, 59 data points (around 48%) were below the 45° line, signifying an equal proportion of over- and under-estimation of the radius values.

These data correspond to the set of experiments at 0.01 mm/min and very similar general observations could be obtained for the tests at 1 mm/min. Note that the data in Figure 4c correspond to E* = 30 GPa. A summary of the mean value, standard deviation (*Stdev*), and the maximum and minimum values from the analysis of local and equivalent radii are given in Table 2 for the set of tests performed at 0.01 mm/min. For a given method of analysis (Matlab or manual), the standard deviation and respected coefficient of variation generally have high values, indicating a scatter in the computed radii, which is expected for natural materials. However, the more precise analysis performed with Matlab also resulted in greater variations in terms of standard deviation and respective coefficient of variation compared with the manual method. Specifically, with respect to the local radius estimation, Matlab and manual analyses gave very similar average value, but the standard deviation values from the Matlab analysis was almost two times larger than that of the manual process, resulting as well in a greater deviation between the maximum and minimum R* values using Matlab. In terms of local radius, slightly greater average *R* (*RB* and *RT*) values were found using Matlab, but (similar to the equivalent radius) the major differences were observed in the standard deviation with a greater span between estimated maximum and minimum values compared with the manual analysis.

When the manual method was adopted to estimate the local radius, the particle picture needed to be enlarged. This may have induced the following issues: (1) the boundary between the particles and the background becoming blurred; (2) the boundary of the particles becoming jagged. In addition, the manual method can be influenced by some subjective judgment. For example, when observing the local radius of relatively flat particles in the contact region, in order to fit the boundary more closely, the manually searched arc’s range is larger than its actual contact range. But when the particle is sharp in the vicinity of the contact, the local radius obtained by the manual method will be smaller than that obtained by computer recognition. This may be due to the fact that when the contact region is extremely small (in the case of grains with corners or sharp edges), it is difficult to clearly determine the boundary of the particles in the enlarged picture, causing measurement errors. When the particle has a fairly regular shape in the vicinity of the contact (x-direction and top particle), the differences between the two methods are expected to be small. The Matlab method can be adopted to avoid the above-mentioned problems, as the images are processed in binary form and estimations based on particle boundary reduce the induced subjectivity in the assessment of particle local shape. However, the resolution of the images plays a significant role in estimating the parameters in both the Matlab and manual methods. The present analyses were performed with images at 640 × 480 pixels or 2133 dpi. Đuriš et al. [43] recommended an optimal resolution of 600 dpi for grains of a few millimeters in size.

**Figure 5:**
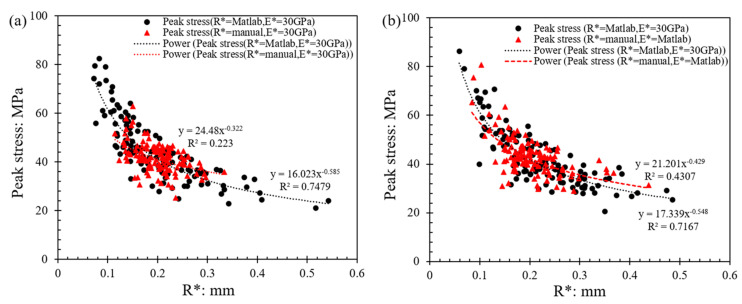
Effect of particle equivalent radius on peak stress at a loading rate of (**a**) 0.01 mm/min; (**b**) 1 mm/min.

In Figure 5, the influence of particle (local) radius on the resultant strength (peak stress) of the grain contacts is presented.

It was observed that the equivalent radius, which is computed from the local radii of the two grains, has a great influence on the peak stress, and Matlab or manual analysis gives the same pattern of decreasing stress at failure with increasing the equivalent radius. Power-law-type fitting was applied, and the respective expressions from both Matlab and manual analyses are displayed in Figure 5a for 0.01 mm/min speed and 5b for 1 mm/min speed. Because of the variations in the computed R*, Matlab analysis resulted in higher absolute values of the exponents (0.585 and 0.548 for 0.01 and 1 mm/min speed, respectively) compared to the manual analysis (0.322 and 0.429 for 0.01 and 1 mm/min speed, respectively). These results suggest a greater sensitivity of the estimated strength on the equivalent radius when Matlab-based analysis is used for image processing compared with that of eyeball fitting. This will also have an influence on the analysis of statistical parameters, as will be discussed in subsequent sections. 

### 3.2. Contact Strength Based on Weibull Analysis

**Figure 6:**
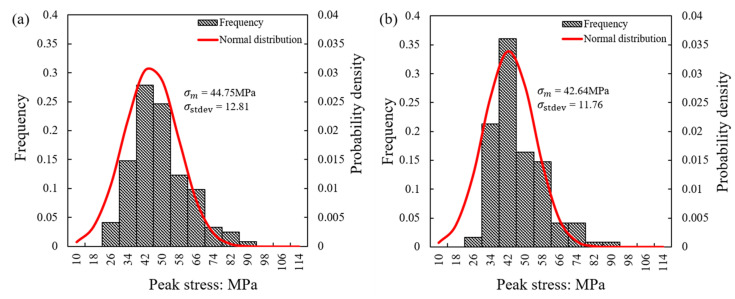
Frequency distribution of peak stress for different loading rates: (**a**) 0.01 mm/min; (**b**) 1 mm/min.

**Figure 7:**
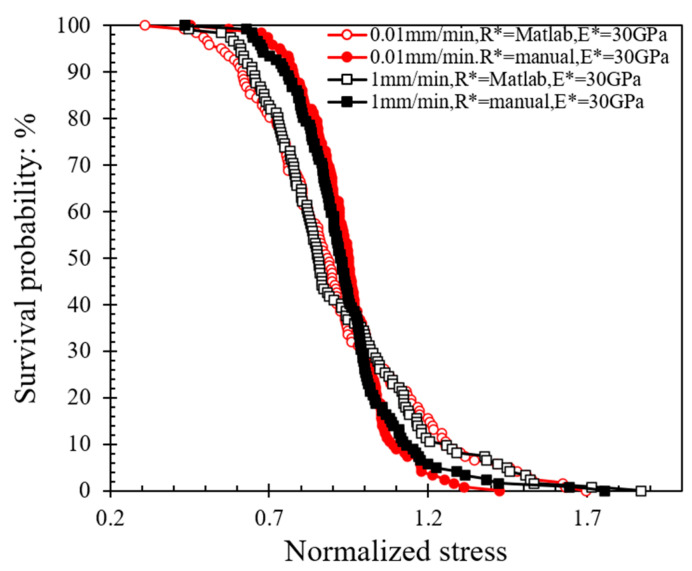
Survival probability against normalized stress curves at different loading rates applying Matlab and manual methods for local morphology analysis.

**Figure 8:**
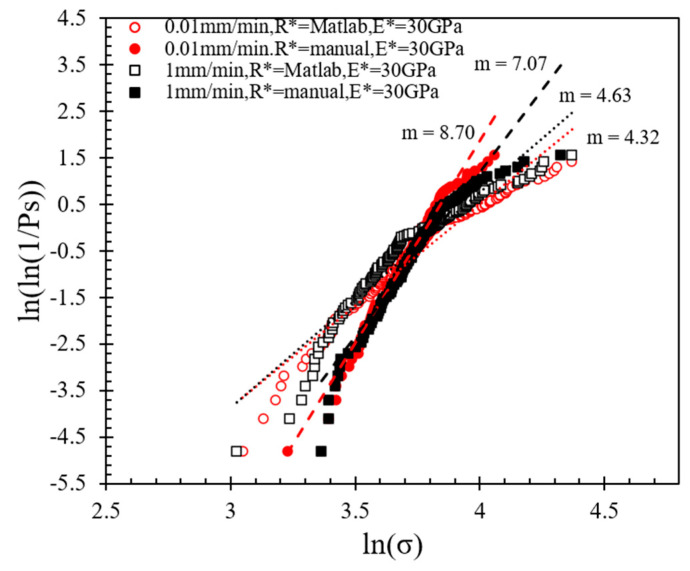
Weibull distribution plots at different loading rates applying Matlab and manual methods for local morphology analysis.

Weibull statistics (after [44]) has often been implemented in the analysis of the crushing behavior of particles [21,22,27,45,46]. Based on the assumption of Hertz contact response and the analytical expressions presented in Section 2.2, the contact strength of the specimens is presented in Figure 6, Figure 7 and Figure 8, where the distribution of peak stress for all the experiments is given in Figure 6 and the corresponding survival probability and Weibull analysis are given in Figure 7 and Figure 8.

The results showing the frequency of the peak stress among the total set of 122 tests for each loading rate in Figure 6 display a normal distribution, expressed through Equation (6).

(6) $$f\left(x\right)=\frac{1}{\sqrt{2\pi }\sigma_{stdev}}exp\left(-\frac{{\left(x-\sigma_{m}\right)}^{2}}{2\sigma_{stdev}^{2}}\right)$$

where *f(x)* is the normal distribution function, *σ_m_* is the mean value of particle tensile stress, and *σ_stdev_* is the standard deviation.

In Figure 6, R* from Matlab analysis and E* = 30 GPa were used to compare the frequencies of the peak strength for the two different speeds. The abscissa on the left side of each sub-figure represents the frequency of the crushing stress in a certain interval. For both speeds, the particle crushing stress was concentrated at 34–66 MPa. At the lower speed, the frequency was 28% at the maximum failure stress, and when the speed increased 100 times, the proportion only rose by 8%, indicating some possible small influence of the loading rate, though within the scatter of the data.

*σ_m_* is the position of the central tendency of the normal distribution, and the value is the abscissa of the peak of the curve. The expected value was 44.8 MPa at 0.01 mm/min and 42.6 MPa at the speed of 1 mm/min. At different loading rates, the probability of breaking peaks close to *σ_m_* is large, and the value farther from *σ_m_* is less likely to occur, which is consistent with the breaking frequency displayed by the bar chart. At the two different loading rates, the values of *σ_stdev_* (standard deviation) were 12.8 MPa and 11.8 MPa, respectively, resulting in a very small coefficient of variation of the two data. The *σ_stdev_* of 0.01 mm/min was slightly larger than 1 mm/min, and the curve of 0.01 mm/min mildly flat.

The survival probability is plotted against the normalized stress in Figure 7; the normalized stress is defined as the ratio of peak stress divided by the characteristic stress (*σ_c_*), which describes the minimum strength that 37% of the population possessed. Based on the data in Figure 7 and the implementation of Weibull analysis, Equation (7a) was used. By applying natural logarithm function on both sides twice, as shown in Equation (7b), as well as sorting the stress data obtained by different methods (from smaller to larger values) to obtain *P_s_*, the log plot of *P_s_* against the tensile strength (Figure 8) can provide the Weibull modulus (*m*).

(7a) $$P_{S}=exp\left[-{\left(\frac{\sigma }{\sigma_{c}}\right)}^{m}\right]$$

(7b) $$ln\left(\ln\left(\frac{1}{P_{S}}\right)\right)=mln\sigma -mln\sigma_{c}$$

where *P_s_* is the survival probability, *m* is the Weibull modulus and *σ_c_* is the characteristic strength.

The data in Figure 8 suggest that the *m*-modulus values obtained by the Matlab method and the manual method for a given speed were significantly different, whereas the influence of speed was relatively small. At a speed of 0.01 mm/min, the *m*-modulus value obtained by the Matlab method was 4.32, but if the peak stress calculated by the manual method were to be used, this set of values would be doubled in magnitude (*m*-modulus equals to 8.70) and the curve would be steeper. A steeper curve implies that the peak stress values (or particle strength values) had a smaller variation when the manual method was applied. In the case of 1 mm/min tests, the changes of *m*-modulus obtained by the two different radius calculation methods followed the same trend, that is, the stress values obtained by the Matlab method had much greater variation than the manual method; implementing the Matlab method, *m*-modulus slightly increased from 4.32 to 4.63, and implementing the manual method, *m*-modulus slightly decreased from 8.70 to 7.07 at speeds of 0.01 to 1 mm/min, respectively. According to Equations (3) and (4), the difference between the stress calculation results of the two methods is mainly because of the resultant variation in R* values (as also elaborated in Section 3.1).

Based on the results from the present study on grain-to-grain systems employing Matlab analysis, the *m*-modulus would be considered as relatively higher in magnitude compared with the reported value (*m*-modulus equal to 3) in the study by Wang and Coop [21] for LBS grains (1.18–2.36 mm) based on single-grain crushing tests and higher compared with the multiple-grain crushing tests reported by Todisco et al. [22] (*m*-modulus in a range of 2.5 to 3.5, independent on the number of contacts based on the latter study). The differences in strength (mean values and range of values) could be considered similar between the grain-to-grain systems in the present study with the single-grain crushing and multiple-grain crushing tests in the studies by [21,22] for LBS particles. It is noted that in both the previous works by Wang and Coop [21] and Todisco et al. [22] the studied particles at failure had a greater degree of freedom (e.g., rotation), which was not allowed in the particle systems examined in the current study. As mentioned earlier, it is expected that within an assembly of grains under in situ conditions the particles may not be necessarily fully free to rotate as they are confined because they are often in contact with many neighboring grains. This means that the single-particle crushing test may not necessarily be the optimum “modeled condition”. On the other hand, the specimens in the present study were fully fixed (i.e., rotational freedom was not allowed), which is again a condition that does not necessarily describe the most “accurate model” for all the grains of a given assembly. It is anticipated that in assemblies of particles, for example, in conditions of approximately one-dimensional compression or in a heavily confined group of proppants in a fracture, a very small degree of freedom against rotation (or an equivalent “fixed system”) may be a more realistic representation. However, discrete-based numerical simulations could give further light to this problem. Note that the main contribution from the aforementioned discussions is not an optimum model in terms of particle boundary conditions, but the critical assessment of different methods of analysis in the implementation of Weibull statistics in particle breakage.

Thus, two major factors influencing the *m*-modulus could be summarized as: (1) the degree of freedom of the grains; (2) the method of analysis used to interpret the crushing tests, i.e., the implementation of the Matlab or manual method (eyeball fitting) in the analysis of the local radius seems to be the most critical factor in calculating the *m*-modulus. The Weibull statistics, both characteristic strength and Weibull modulus, can be primarily used to compare the strength characteristics of different materials, and they have been used as parameters to correlate the crushing strength and size of sand grains [47]. The proportionality between the tensile strength and size of the grains was observed to be based on the Weibull modulus [48], but the correlation might not hold good for every material. For example, Nakata et al. [49] studied the crushing behavior of silica sand using Weibull statistics, but the Weibull modulus did not show regular trends with the proportionality between characteristic strength and size of the grains. However, McDowell [47] suggested the use of average crushing strength to be related to grain size based on the Weibull modulus. These issues might also be related to the degree of freedom of a particle in a given assembly of grains, as the single-particle crushing model, which is often adopted to interpret the behavior of bulk laboratory or numerical samples, may not be necessarily the optimum reference model.

### 3.3. Implementation of Hertz Analysis of the Force–Displacement Curves to Derive Contact Young’s Modulus and Failure Stress

In the data analysis presented in the previous sections, Hertz response was assumed to estimate the contact radius (*α*), from which the peak stress (or stress at failure) was calculated based on Equations (2)–(4). As described in Section 2.2, E* was assumed to be 30 GPa for all these analyses. This may be a representative average value expected for LBS grains, however, each different specimen may display deviations from the pre-assumed value of 30 GPa, as has also been discussed in previous studies [24,25,42]. Thus, an attempt was made to examine the differences in the observed failure strength and respected *m*-value from the Weibull analysis by analyzing each individual experimental (force–displacement) curve to derive a specific contact modulus (E*) value for each of the different pairs of grains. For this purpose, Hertz expressions from Equations (2) and (3) were analyzed in conjunction with Equation (8), in which case E* is used as the model fitting parameter to match the theoretical (Hertzian) curve to the experimental data.

(8) $$F=\frac{4}{3}{\left(R^{*}\right)}^{0.5}E^{*}\delta^{1.5}$$

where *δ* and *F* are the displacement and load, respectively, during the crushing test.

**Figure 9:**
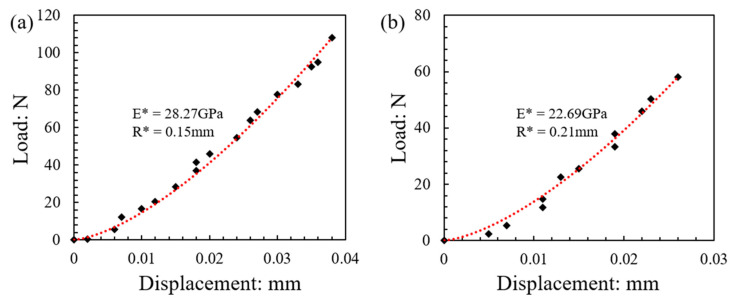
(**a**,**b**) Two examples of Hertz fitting to the load–displacement curves to estimate contact Young’s modulus (corresponding to 0.01 mm/min speed).

**Figure 10:**
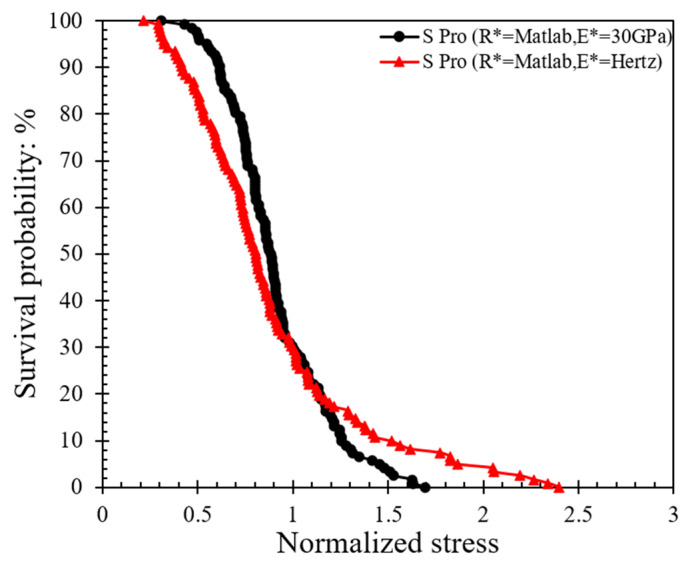
Effect of equivalent modulus on the survival probability at 0.01 mm/min speed.

**Figure 11:**
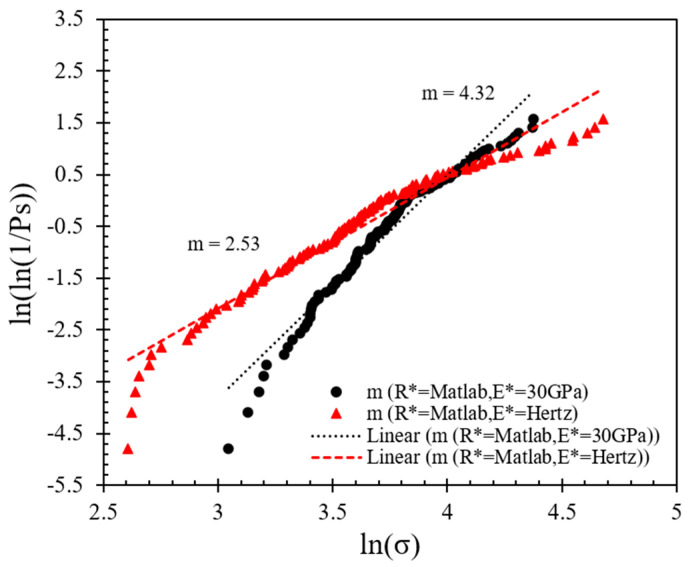
Weibull distribution plots for different equivalent moduli at 0.01 mm/min speed.

Examples of Hertz model implementation to the experimental force–displacement curves are shown in Figure 9 (corresponding to 0.01 mm/min speed). Based on this analysis for the whole set of 122 tests at 0.01 mm/min loading rate, the survival probability against the normalized stress and respective Weibull characteristics are plotted in Figure 10 and Figure 11 (similar to the analysis presented in Figure 7 and Figure 8). The plots also provide a comparison between the survival probability curves and Weibull modulus values related to Hertz analysis with E* obtained for each test and E* = 30 GPa case for all the tests. Note that in the plots of Figure 10 and Figure 11 all the data correspond to Matlab analysis for the estimation of the local and equivalent radii (*RB*, *RT*, and R*) and Hertz fitting was applied only for the tests at 0.01 mm/min (as the faster loading rate of 1 mm/min resulted in fewer data points and thus the fitting could not be considered as reliable).

By analyzing each load–displacement curve with the Hertz model (for 122 curves), it was found that E* ranged, approximately, from 6.7 to 53 GPa, with an average value and a standard deviation of 25.7 ± 9.3 GPa. The average value from this fitting process is close to the assumed value of 30 GPa that was implemented in the previous analyses, and the coefficient of variation (i.e., the ratio of standard deviation to the mean value) corresponded to 36%, which shows a general agreement with the reported data by Sandeep and Senetakis [24]. However, it is acknowledged that within the total set of 122 tests at 0.01 mm/min speed, a great scatter in the data can be observed, as also implied from the minimum and maximum obtained E* values. 

Based on the fitting of each experimental curve using the Hertz model, the re-analysis of the data as presented in Figure 10 and Figure 11 suggests that the *m*-modulus is smaller in magnitude compared with the assumption of E* = 30 GPa for all the pairs of grains, which implies a greater variation in the strength characteristics of the LBS grain contacts.

Note that the implementation of the Hertz model in the complete analysis of the results (i.e., the calculation of contact radius and Young’s modulus) in conjunction with Matlab analysis for the local radius provided an *m*-value that is much closer to the reported results by Wang and Coop [21] for single-grain crushing tests. Similar to the discussions in the previous sections, these data would suggest that the most critical factor in the assessment of the strength characteristics of soil particles is the method of analysis adopted (assuming contact Young’s modulus as a constant value for all the specimens or its calculation is performed for each individual specimen) has an impact on the interpretations from the Weibull analysis.

### 3.4. Clustering Analysis

An investigation into the relationship between failure stress with contact modulus (E*), equivalent radius (R*), and maximum displacement (i.e., displacement corresponding to peak load) was conducted in an attempt to examine any possible consistent patterns of behavior from the grain-to-grain crushing tests. In this case, clustering was applied using the Microsoft Excel software’s plugin XLSTAT program for data analysis. The application of clustering and other artificial intelligence techniques has been adopted for geotechnical site investigation research [50] and more primarily in surface characterization applications such as hardness measurements of geological materials [51,52]. Considering the classification problem that requires unlabeled learning, the Gaussian mixture model was selected in this analysis; the Gaussian mixture model can be represented by Equation (9).

(9) $$p\left(x\right)=\overset{K}{\underset{k=1}{\sum }}\pi_{k}N\left(x|\mu_{k},\Sigma_{k}\right)$$

where $\pi_{k}$ is the mixture coefficient that can be regarded as the weight of each $N\left(x|\mu_{k},\Sigma_{k}\right)$; $\mu_{k}$, and $\Sigma_{k}$ are the mean and co-variance values.

**Figure 12:**
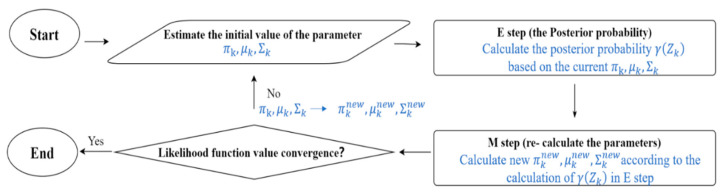
Flowchart showing the clustering analysis performed in the present study.

When performing clustering analysis on E*, R*, and maximum displacement, according to the Bayesian information criterion, the best mixture model is the VVV (variable volume, variable shape, variable orientation), and the EM (expectation maximization) algorithm converged in 79 iterations was used to obtain the parameters of the Gaussian distribution function model and the probability of the model. A flowchart of the process followed in the present study is given in Figure 12. 

**Figure 13:**
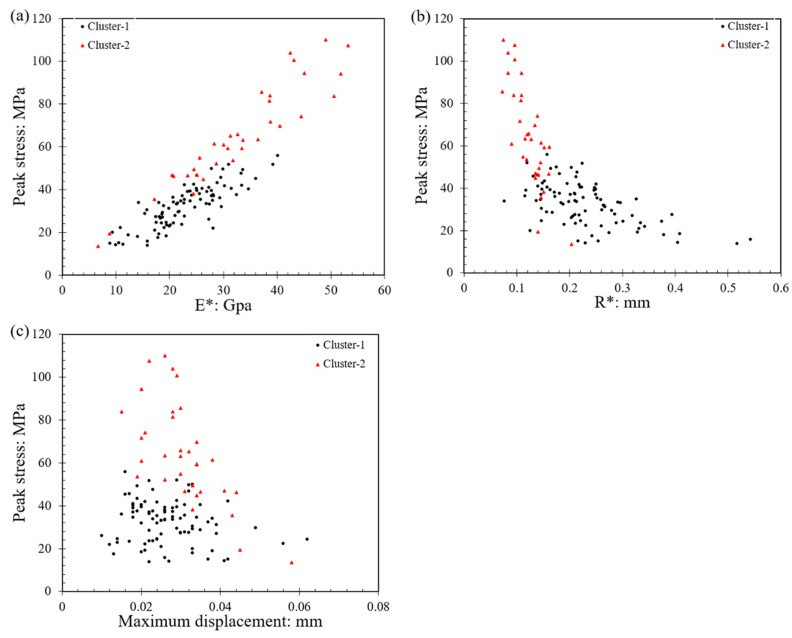
Clustering analysis investigating (**a**) the relationship between the equivalent modulus and the peak stress, (**b**) the relationship between the equivalent radius and the peak stress, (**c**) the relationship between the maximum displacement (corresponding to peak load) and the peak stress.

The optimum classification of the clusters is two classes, of which cluster-1 is the predominant one, accounting for 72% of the data points. In Figure 13a, the distribution of cluster-1 points is concentrated in the lower regime of the equivalent modulus (E*) values. The equivalent radius (R*) of the data points with higher peak stress was concentrated in 0.07–0.21 mm and the distribution range was narrower than cluster-1, as presented in Figure 13b. The average E* and R* values of cluster-1 were 22.94 GPa and 0.232 mm, respectively, and the respected values for cluster-2 were 32.36 GPa and 0.125 mm, respectively. For the maximum displacements (i.e., displacements corresponding to peak stress), the values had a similar range between the two clusters (note that the average value of maximum displacements was equal to 0.027 mm for cluster-1 and 0.030 mm for cluster-2. Although there were some overlaps between the data points between the two clusters, the differences between them can still be clearly distinguished as they had very different ranges of peak stress. Cluster-1 represents the data points with the lower equivalent modulus (E*), larger equivalent radius (R*), and lower peak stress. Cluster-2 shows the exact opposite trend compared to cluster-1 data points, with increasing equivalent modulus (E*) and decreasing equivalent radius (R*).

### 3.5. Failure Modes

#### 3.5.1. General Observations and Classification of Breakage Patterns

**Figure 14:**
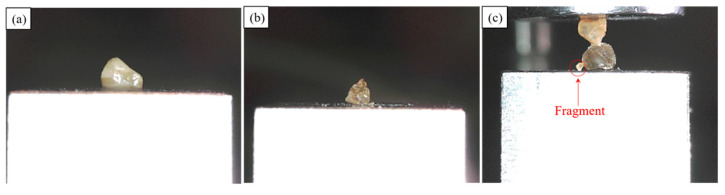
Representative examples of the different failure modes (**a**) conservative, (**b**) destructive, and (**c**) fragmentary.

Three different failure modes were identified from the whole set of 244 experiments in the study. The first mode is termed as “conservative” (C), in which case the grain (either the top or bottom grain) remained intact as a whole body, but some debris, due to the crushing of micro-asperities, could be identified at the contact of the grains after the completion of the test. The second mode of crushing observed is termed as “fragmentary” (F), in which case the main body of the grain remains intact, but some small fragments are observed after the completion of the test. Finally, a third observed mode is termed as “destructive” (D), in which case the particle breaks into two (or more) pieces with a clear fracture developed in the whole body of the grain. Representative examples of these different modes are given in Figure 14.

These three modes of failure (conservative, fragmentary, destructive) correspond to “micro-scale”, “meso-scale”, and “macro-scale” breakage patterns, respectively. Based on this distinction, a total of six different combinations could occur based on the mode of failure of top and bottom grains for each specimen. Examples can be given as: one grain fails in conservative mode and the other grain fails in destructive mode (C-D combination); one grain fails in conservative mode and the other grain in fragmentary mode (C-F combination); both grains fail in destructive mode (D-D combination).

**Figure 15:**
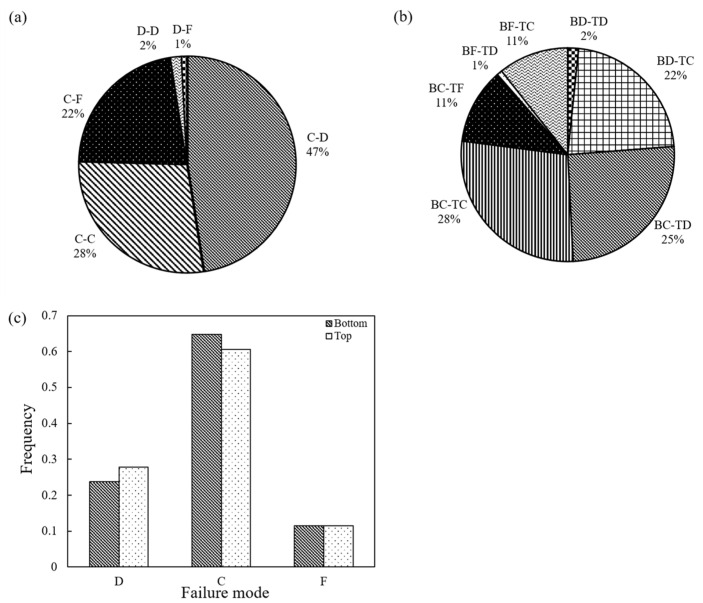
Failure pattern for loading rate 0.01 mm/min: (**a**) system failure mode; (**b**) system failure mode when considering particle position; (**c**) failure mode for top and bottom grains.

With respect to the experiments performed at 0.01 mm/min (low speed), Figure 15a presents the distribution of the different combinations of failure (without distinguishing the specific mode of the top and bottom particles), in which case it is seen that the majority of the experiments had a “C-D” type of failure (47%), while “C-C” or “C-F” types of failure constituted 28% and 22% of the tests, respectively. Thus, in 97% of the tests at 0.01 mm/min speed (118 out of 122 specimens), at least one of the two particles of the whole set of specimens had a conservative mode of failure. The statistics of the modes of failure in terms of the three observed patterns are also represented with the bar graphs of Figure 15c. Finally, a very small portion of the experiments had a D-D or D-F mode of failure, which implies that some meso-scale or macro-scale breakage occurred only in one of the two grains, and a micro-scale failure mode was prevalent in the experiments. This may have important implications in discrete-based modeling of the failure behavior of granular assemblies, as currently most researchers utilize the mode of failure as derived from single-grain crushing tests in the calibration process of numerical samples, whereas at the failure point of a grain system, different neighboring particles may have different breakage modes, as the present results would suggest. This means that when a particle breaks with a destructive mode (complete failure of the whole body of the grain), neighboring grains may also have some damage, for example of the conservative mode, thus the grain breakage is not a problem that should be seen for “isolated” grains but in particle systems of groups of grains.

In general, for the low-speed tests, a specific pattern was not observed for these different combinations, i.e., for the experiments with a “C-D” mode of failure. In approximately half of the cases, the bottom grain had a conservative mode and the top grain had a destructive mode, and vice versa, and the same observation could be drawn from the experiments with a “C-F” mode of failure. The distribution of the modes of failure taking into account how the bottom and top grains failed is given in Figure 15b (where “B” means the bottom particle and “T” means the top particle, thus “BD-TC” means that the bottom grain had a destructive failure and the top grain had a conservative failure).

**Figure 16:**
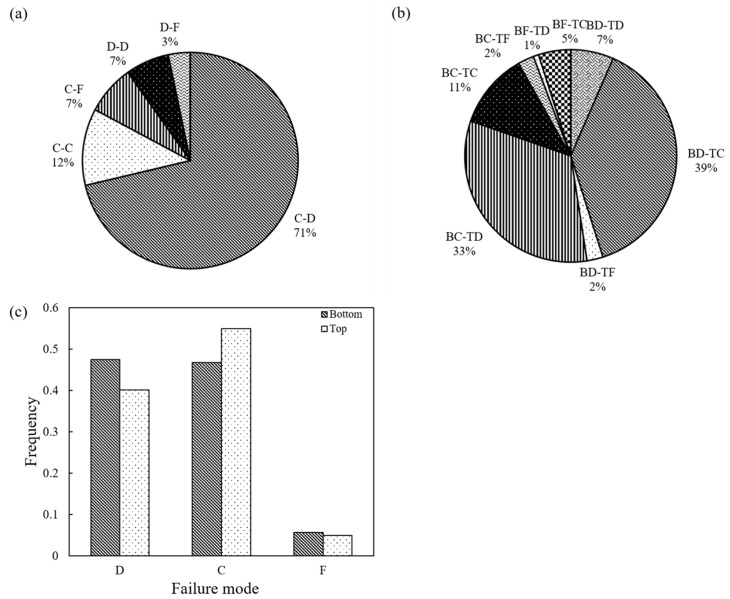
Failure pattern for loading rate 1 mm/min: (**a**) system failure mode; (**b**) system failure mode when considering particle position; (**c**) failure mode for top and bottom grains.

**Figure 17:**
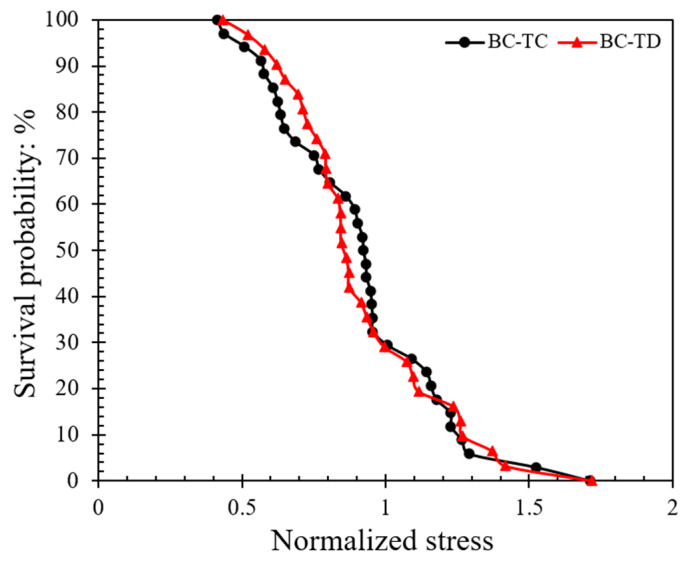
Comparison between different system failure modes on the survival probability for loading rate 0.01 mm/min.

**Figure 18:**
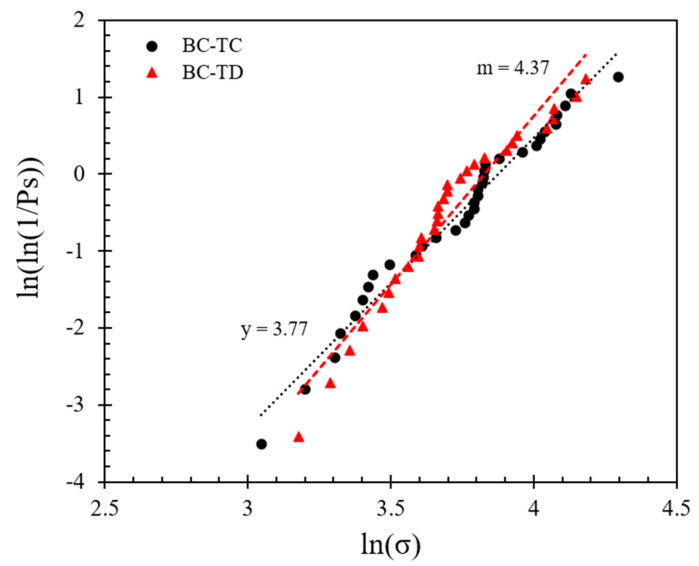
Comparison between different system failure modes on the Weibull distribution for loading rate 0.01 mm/min.

In the tests at higher speed (1 mm/min), the dominant failure mode was C-D (conservative-destructive), taking up 71% of the total set of experiments, and the respective distributions of the different failure combinations for this class of tests are given in Figure 16. At both speeds, a prevalent mode of one of the two grains was conservative, taking up 90% of the tests performed at 1 mm/min (i.e., in 90% of the tests, at least one grain had a conservative failure, which is close to the 97% observed in the tests at 0.01 mm/min). A major difference between the tests at 0.01 and 1 mm/min is that at the lower speed there was an occurrence of a higher portion of tests where one particle had a fragmentary mode (23%), while only in 10% of the tests at 1 mm/min a fragmentary mode was observed. This makes for a slightly different distribution of the failure mode combinations by comparing Figure 15c and Figure 16c. Despite these differences, the mode of failure, in general, did not significantly influence the survival probability. Representative examples comparing the strength characteristics for two of the dominant modes of failure at 0.01 mm/min (BC-TC versus BC-TD) are given in Figure 17 and Figure 18.

As discussed by Wang and Coop [21], from their single-particle crushing tests, they classified the condition of the broken particles into four failure modes, including splitting, explosive, explosive-splitting, and chipping, based on the number of the fragments and the propagation of the cracks (though splitting and explosive were generally the two major modes). Using a high-speed camera, it is technically feasible to distinguish whether the particles split into several large pieces along the internal cracks under the action of normal load or if the particles burst into tiny fragments via instant blasting. In the present tests, the condition of the particles after being broken may have been restricted by the glue, and some crushing types such as splitting or explosive may have overlapped. As the present study examined grain-to-grain crushing tests, the formation of powder in the contact region was a highly possible scenario, as also the data would suggest. In 98% and 93% of the tests at 0.01 and 1 mm/min speeds, respectively, at least one of the two grains did not have macro-scale breakage.

#### 3.5.2. Influence of Breakage Patterns on the Load–Displacement Curves

**Figure 19:**
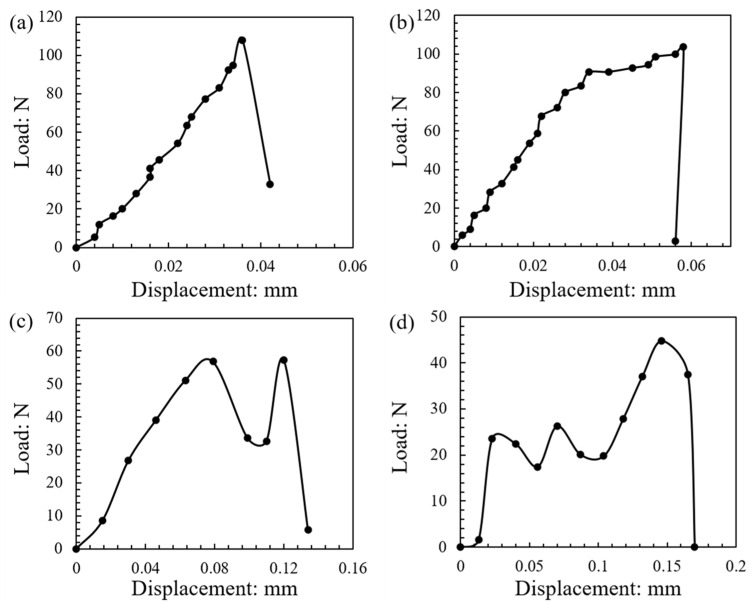
Different classes (types) of load–displacement curves: (**a**) class-A; (**b**) class-B; (**c**) class-C; (**d**) class-D.

**Figure 20:**
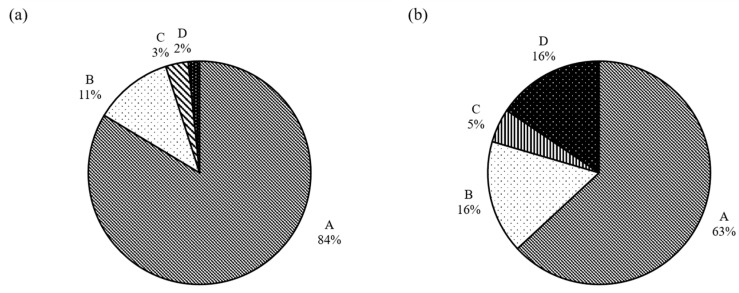
Distribution of the different classes of load–displacement curves for different loading rates: (**a**) 0.01 mm/min; (**b**) 1 mm/min.

In general, four different classes of curves, based on their shape and the occurrence of one peak or multiple peaks, could be distinguished from the total set of experiments. The majority of the load–displacement curves are represented by the Figure 19a, in which case a gradual increase of the load was observed with increasing displacement (assumed to be Hertzian despite some fluctuations in the data points) up to the breakage point (defining the peak force and peak stress). This type of curve represents a “brittle” type of failure, even though the specific pattern displayed some variations, as discussed in Section 3.5.1. This type of initially “approximated” Hertzian response followed by a brittle failure with a single peak point represented 84% of the tests at 0.01 mm/min and 63% of the tests at 1 mm/min, as shown in the statistical distributions in Figure 20 (termed as “class-A”). A second class of load–displacement curves (termed as “class-B”) was again of brittle type, however, after the initial regime of Hertzian response, a hardening behavior was observed, leading, in general, to a shift of the peak stress to larger displacements. This type of behavior (represented in Figure 19b) was observed for 11% and 16% of the tests at 0.01 and 1 mm/min, respectively. For a very small number of tests, two additional classes of load–displacement curves were observed, and representative examples are given in Figure 19c,d, termed as “class-C” and “class-D”. In these curves, it could be observed that after reaching a peak point, a significant drop of the load was observed followed by a second increase with a new peak value close to the previous peak value, while in class-D there was no less than one fluctuation (the value of the previous fluctuation(s) was much smaller than the failure point reached) of the curve at the initial regime of displacements and the peak stress was reached at much larger displacements. 

Even though curves of class-C and class-D counted for only 5% of the tests at 0.01 mm/min, a much larger portion of 16% of class-D curves was observed in the tests at 1 mm/min, which suggests that a higher speed may lead to some increased observations of the fluctuations in the load–displacement curves. This could be related, perhaps, with some influence of creep or inertial effects, but in order to make firm conclusions, this issue should be further explored in future studies by performing tests on a wider range of velocities (note that the experimental setup used in the present study has some limitations on the upper and lower bounds of the applied velocities).

For the experiments at 0.01 mm/min speed, the majority of the tests corresponding to C-C or C-F modes of failure belonged to class-A curves (i.e., brittle behavior with single peak stress without the occurrence of hardening prior to reaching the crushing point). However, failure modes C-C and C-F (represented majorly by class-A of the force–displacement curves) accounted for a much smaller portion of the tests at the higher speed of 1 mm/min. Class-B curves represented, predominantly, samples with failure mode C-D and some tests with mode C-C (i.e., destructive mode of one of the two grains was generally accompanied by a behavior characterized by a hardening regime prior to reaching the crushing point, but it should be noted that this was not the case of 100% of curves in class-B). Class-D, which occurred predominantly in the experiments at 1 mm/min speed, represented most of the tests with at least one particle having a destructive mode of failure (C-D, D-D, D-F combinations). However, as creep (or loading rate) influences might come into play in the experiments, it is considered that future studies could further explore the correlation between failure mode and class (or shape) of the load–displacement curves to provide clearer conclusions in this regard.

**Figure 21:**
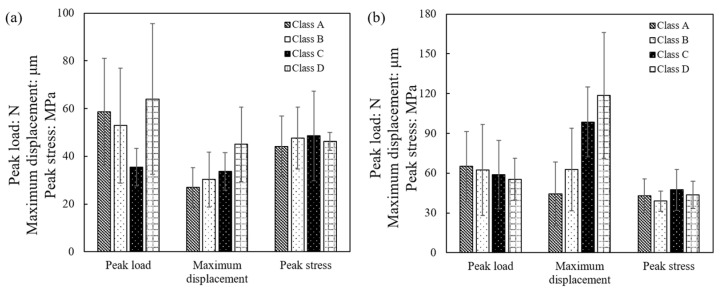
Peak load, maximum displacement (corresponding to peak load) and peak stress for the different classes of curves at velocities of: (**a**) 0.01 mm/min; (**b**) 1 mm/min.

Figure 21 gives, in the form of bar graphs, the correlation of peak load, displacement corresponding to the peak load, and the peak stress with the type of curve (class-A to class-D). The bar graphs indicate mean values and the standard deviation values are also indicated in these plots. Despite the scatter in the data, it can be observed that the maximum displacement increased from class-A to class-D, however, for each different velocity, firm conclusions could not be drawn on the influence of the shape of the load–displacement curve on the peak stress. 

## 4. Conclusions

Based on the grain-to-grain crushing tests in the present study, the following major conclusions, new contributions and recommendations for future research are summarized:

(1) The analysis of the data showed that even though the grain-to-grain systems in the present study had a similar range of strength (peak stress) with that of single- and multiple-contact crushing tests as presented in the literature, *m*-modulus values were, in general, higher in magnitude in the present work. This leads to the conclusion that the restriction of the grains to rotate during their compression leads to a smaller variability of peak stress values, even though it did not influence greatly the absolute values of strength. This may have important implications in integrating laboratory grain-scale tests with discrete-based numerical simulations, as currently the majority of numerical works implement in their calibration results from single-grain crushing tests. However, under in situ conditions, it is not necessary for the particles to be represented by the single-grain crushing test model.
(2) The method used to estimate the contact radius and the equivalent radius, thus the contact area (by applying Hertz expressions), heavily influenced the calculated *m*-modulus. In general, the image processing using Matlab to compute the local radius in the vicinity of grain’s contacts led to much lower values of *m*-modulus compared with a manual estimation based on the obtained digital images. Despite that, the computed equivalent radius had a significant influence on the resultant peak stress, though this influence was more pronounced for the data where Matlab analysis was used compared with the manual method. It is noted that the manual method is not conceptually incorrect, in that the user defines, based on a digital image, a circle in the vicinity of the particle peak (where the contact with the neighboring grain is expected to occur), through which the local morphology is assessed. However, the Matlab analysis can capture more precisely the small differences in the morphologies of different grains, and this is very important to be considered in natural materials that display irregular shapes. The manual method homogenizes to some extend the assessed morphological characteristics of natural grains, giving rise to increased *m*-modulus values that inherently represent the variation of the characteristics within a population. Previous studies examining the breakage of grains have considered more “global” shapes rather than the locality of the particles, which, based on the present study, may lead to inaccurate estimation of the failure stress (as the failure stress is influenced by the estimated contact area).
(3) The application of the Hertz model was implemented in two different steps. First, it was used to estimate the contact area to derive stresses from the measured forces; second, it was used to compute the contact Young’s modulus (E *) of each individual sample. Implementing the Hertz model to compute E * for each individual test had an influence on the resultant peak stress compared with the manual method (eyeball fitting), in which E * was assumed to be the same (30 GPa) for all the pairs of grains. This means that in the analysis of the breakage of particles, assessment of the elastic (and morphological) characteristics of the given sample is desirable.
(4) Despite the significant differences in the estimated peak stress based on the method of analysis used, experiments at two different speeds (0.01 and 1 mm/min) provided similar results in terms of peak stress and *m*-modulus values. However, the speed of the tests had some influence on the mode of failure of the samples and also on the shape of the force–displacement curves. As the speed in the present study was examined within two folders, it is recommended that in future research works a wider range of velocities could be applied to examine the problem of grain breakage.
(5) In general, the majority of the tests had a conservative-destructive or conservative mode of failure, where conservative implies micro-scale damage and formation of debris in the vicinity of grain contacts, while destructive implies macroscopic grain breakage. In some of the tests, a fragmentary mode of failure was observed, in which case meso-scale breakage and formation of small fragments occurred. A greater portion of fragmentary-type failure was observed at a higher speed. Thus, in the calibration of discrete-based numerical samples, it is important to consider how grains interact with respect to their breakage behavior rather than implementing the modes of failure as single-grain crushing tests would suggest (i.e., at the breakage point, a particle should not be seen as an isolated body, but its interaction with neighboring grains must be taken into account).
(6) Four classes of force–displacement curves were distinguished, in which case most of the samples had a brittle failure with clear peak stress (with or without the occurrence of hardening prior to reaching peak stress). In some of the tests, more prominently at the higher speed, multiple peaks were observed with a significant shift of the displacements (corresponding to peak stress) to higher values. This behavior was ascribed to possible creep influences; however, it is recommended that future studies could further explore this behavior to draw firm conclusions.

## Figures and Tables

**Table 1:** Summary of tests and respective analysis.

Materials: Leighton Buzzard Sand (LBS); Size: 1.18–2.36 mm				
Loading Rate	Number of Tests	Test for Radius		Hertz Fitting
		Manual	Matlab	
0.01 mm/min	122	•	•	•
1 mm/min	122	•	•	

**Table 2:** Summary of test results in terms of local radius and equivalent radius computation based on Matlab and manual analysis of local morphology.

	Local Radius (*R*): mm					Equivalent Radius (R*): mm			
	Position	Mean	Stdev	Max	Min	Mean	Stdev	Max	Min
Matlab	Bottom	0.46	0.22	1.34	0.11	0.2	0.09	0.54	0.07
	Top	0.44	0.25	1.49	0.09				
Manual	Bottom	0.42	0.12	0.94	0.16	0.2	0.04	0.33	0.11
	Top	0.41	0.12	0.83	0.17				

## Data Availability

Data are available from the corresponding author upon reasonable request.
